# The Microbacillus of Acne

**Published:** 1910-10-08

**Authors:** 


### Dermatology.

### THE MICROBACILLUS OF ACNE.

The normal skin abounds in staphylococci, so that when pus inoculation develops upon the top of any other skin lesion it is not at all surprising that the organism most often recovered is the staphylococcus. It is always very difficult to be sure that this is the organism which actually causes the lesion in the first instance, and this has been a particular difficulty in connection with ordinary acne vulgaris. It has, however, been established almost beyond doubt that the primary organism in acne is a bacillus, so that in the vaccine treatment of the disease, even though anti-staphylococcal vaccine may be required in the relief of acute pustulation, a vaccine prepared from the acne microbacillus is also required if the acne itself is to be materially benefited. A great deal has been written about the media required for the cultivation of this organism, and various special reagents have been employed. The results have been very various at the hands of different observers, and until recently it has not even been firmly established whether the bacillus is aërobic or anaërobic. Recent work, however, has shown that the secret of success in cultivating the organism is the use of anaërobic methods when even so simple and easily prepared a medium as 2 per cent. glucose agar gives luxuriant growth.

